# Proliferation of *Aedes aegypti* in urban environments mediated by the availability of key aquatic habitats

**DOI:** 10.1038/s41598-020-69759-5

**Published:** 2020-07-31

**Authors:** André Barretto Bruno Wilke, Chalmers Vasquez, Augusto Carvajal, Johana Medina, Catherine Chase, Gabriel Cardenas, John-Paul Mutebi, William D. Petrie, John C. Beier

**Affiliations:** 1 Department of Public Health Sciences, Miller School of Medicine, University of Miami, 1120 Northwest 14th Street, Miami, FL 33136 USA; 2 Miami-Dade County Mosquito Control Division, Miami, FL USA; 3 Division of Vector-Borne Diseases, Centers for Disease Control and Prevention, Fort Collins, CO USA

**Keywords:** Ecology, Ecological epidemiology, Urban ecology

## Abstract

*Aedes aegypti* is the main vector of dengue, Zika, chikungunya, and yellow fever viruses. Controlling populations of vector mosquito species in urban environments is a major challenge and being able to determine what aquatic habitats should be prioritized for controlling *Ae. aegypti* populations is key to the development of more effective mosquito control strategies. Therefore, our objective was to leverage on the Miami-Dade County, Florida immature mosquito surveillance system based on requested by citizen complaints through 311 calls to determine what are the most important aquatic habitats in the proliferation of *Ae. aegypti* in Miami. We used a tobit model for *Ae. aegypti* larvae and pupae count data, type and count of aquatic habitats, and daily rainfall. Our results revealed that storm drains had 45% lower percentage of *Ae. aegypti* larvae over the total of larvae and pupae adjusted for daily rainfall when compared to tires, followed by bromeliads with 33% and garbage cans with 17%. These results are indicating that storm drains, bromeliads and garbage cans had significantly more pupae in relation to larvae when compared to tires, traditionally know as productive aquatic habitats for *Ae. aegypti*. Ultimately, the methodology and results from this study can be used by mosquito control agencies to identify habitats that should be prioritized in mosquito management and control actions, as well as to guide and improve policies and increase community awareness and engagement. Moreover, by targeting the most productive aquatic habitats this approach will allow the development of critical emergency outbreak responses by directing the control response efforts to the most productive aquatic habitats.

## Introduction

The prevalence and incidence of mosquito-borne viral diseases are increasing globally. Dengue is of great public health concern currently occurring in 128 countries^1–4^, and more than 1 million cases of Zika virus and its associated microcephaly and fetus malformation were reported in the Americas between 2016 and 2017^5–8^. Despite the availability of an effective vaccine^9–14^, a major yellow fever virus outbreak was reported in Brazil^15^ and across Africa representing a heavy toll for countries like Angola and the Democratic Republic of the Congo in Africa^16–18^. Therefore, due to the lack of effective treatments, controlling populations of vector mosquito species is considered the most effective method to prevent the transmission of arboviruses to humans^19–21^. *Aedes aegypti* is the main vector of dengue, Zika, chikungunya, and yellow fever viruses in urban areas^22^. It is widely distributed in the tropical and sub-tropical regions of the world and is well adapted to thrive in urban environments^23–29^, where there is increased contact between mosquito vectors and human hosts, thus facilitating arbovirus transmission^30^.

Controlling populations of vector mosquito species in urban environments is a major challenge. *Aedes aegypti* is well adapted to and will successfully exploit many artificial and natural habitats present in urban environments, presenting a major challenge for the development of control strategies^31^. Reactive control strategies based on the use of larvicide and adulticide are widely ineffective due to the inherent difficulty in reaching cryptic breeding habitats and resting adult mosquitoes^32^. Moreover, *Ae. aegypti* populations have high levels of insecticide resistance which will further impair the effectiveness of reactive mosquito control strategies in urban environments^33,34^.

Alternative vector control strategies such as the release of genetically modified or *Wolbachia* infected mosquitoes are still years away from being used in real-world operations and their effectiveness in controlling not only mosquito populations but also in decreasing the incidence of arbovirus transmission is yet to be proven^35,36^. Controlling populations of vector mosquitoes in urban areas is a difficult task and control strategies based on the Integrated Vector Management (IVM) framework are complex relying on many actions that rationally build on each other. However, the IVM key components such as mosquito surveillance, source reduction (i.e., aquatic habitat removal), community engagement, and improved policies can achieve great success^20^.

Therefore, being able to determine the role of the aquatic habitats that are widely present in urban areas and are responsible for maintaining *Ae. aegypti* populations will allow not only the development of more effective preventative mosquito control strategies but would also help guide and improve policy and better inform the community^37–39^. We hypothesize that among all the potential aquatic habitats present in urban areas of Miami-Dade County, Florida that are suitable for *Ae. aegypti*, a few key aquatic habitats are responsible for the majority of the proliferation of *Ae. aegypti*. Therefore, our objective was to leverage on the immature mosquito surveillance system based on requested by citizen complaints through 311 calls^31^ to determine what are the most important aquatic habitats in the proliferation of *Ae. aegypti* in Miami and which aquatic habitats should be prioritized in mosquito management and control strategies.

## Results

From a total of 3,354 household inspections, *Ae. aegypti* was found in 2,590 households totaling 17,822 larvae and 3,402 pupae. From all of the many aquatic habitats in which *Ae. aegypti* was found breeding in, approximately 80% of all *Ae. aegypti* collected were found breeding in nine aquatic habitats: bromeliads, buckets, plastic containers, flower pots, fountains, garbage cans, planters, storm drains, and tires. Bromeliads were the most commonly inspected breeding habitat totaling 739 inspections, followed by storm drains, and buckets totaling 452 and 363 inspections, respectively. On the other hand, the less commonly inspected breeding habitat were garbage cans totaling 75 inspections, followed by planters totaling 70 inspections.

*Aedes aegypti* was most commonly found in bromeliads, buckets, and flower pots, considering both the total number of larvae and pupae. However, the average number of *Ae. aegypti* collected per aquatic habitat varied greatly. Flower pots and buckets yielded an overall higher average of immature mosquitos (larvae and pupae), with an average of 16, 14, and 12 specimens per inspection, respectively. On the other hand, bromeliads yielded a lower average of 5 specimens per inspection, and storm drains yielded an average of 2 (Table 1).

**Table 1:** Number of immature *Aedes aegypti* collected in Miami-Dade County, Florida, from June 1st, 2018 to October 31st, 2019 by habitat.

Habitats	Inspections	Larvae	Pupae	Total	Average *Aedes aegypti* collected per inspection	Average Larvae	Average Pupae
Bromeliads	739	2,488	847	3,335	5	3	1
Buckets	363	3,846	618	4,464	12	11	2
Flower Pots	281	3,484	560	4,044	14	12	2
Fountains	188	1,628	240	1,868	10	9	1
Garbage cans	75	639	121	760	10	9	2
Planters	70	544	93	637	9	8	1
Plastic containers	158	1,581	242	1,823	12	10	2
Storm drains	452	642	341	983	2	1	1
Tires	229	2,468	272	2,740	12	11	1

The tobit model revealed that storm drains had a 45% lower percentage of *Ae. aegypti* larvae over the total of larvae and pupae adjusted for daily rainfall when compared to tires, followed by bromeliads with 33% and garbage cans with 17% lower percentage of *Ae. aegypti* larvae over the total of larvae and pupae adjusted for daily rainfall when compared to tires (Table 2).

**Table 2:** Tobit regression model of the percentage of *Aedes aegypti* larvae over the total of larvae and pupae adjusted for daily rainfall in Miami-Dade County, Florida, from June 1st, 2018 to October 31st, 2019 by habitat.

Habitats	Estimate	Standard error	Significance *P*
Intercept	0.994814	0.04234	**< 0.0001**
Rainfall	− 0.057539	0.02833	**0.0422**
Bromeliads	− 0.339891	0.04741	**< 0.0001**
Buckets	0.041822	0.05354	0.4348
Plastic Containers	− 0.045725	0.06541	0.4845
Flower Pots	− 0.010125	0.05634	0.8574
Fountains	0.00686	0.06206	0.912
Garbage Cans	− 0.176147	0.08135	**0.0304**
Planters	0.0135	0.08615	0.8755
Storm Drains	− 0.456627	0.05026	**< 0.0001**
Tires	0		
Sigma	0.563806	0.01285	**< 0.0001**

Significant values (*P* < 0.05) are highlighted in bold.

**Figure 1:**
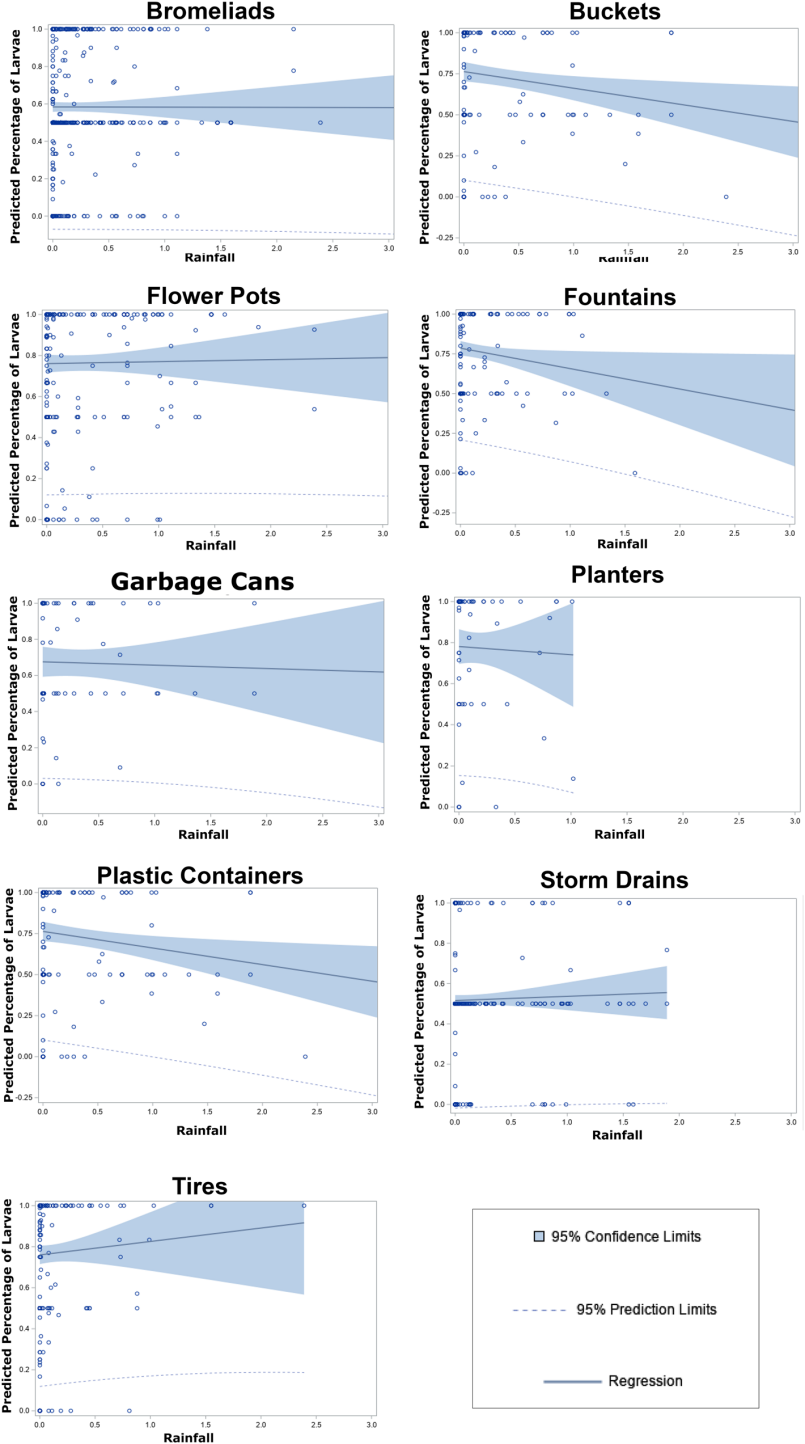
Robust regression estimates of the linear association between daily rainfall and the percentage of *Aedes aegypti* larvae over the total of larvae and pupae within aquatic habitats in Miami-Dade County, Florida, from June 1st, 2018 to October 31st, 2019.

The results from the robust regression to investigate the association between daily rainfall and the decrease in the percentage of *Ae. aegypti* larvae over the total of larvae and pupae by aquatic habitat revealed a significant association between daily rainfall with the percentage of *Ae. aegypti* larvae over the total of larvae and pupae in plastic containers (*P* = 0.0002) and fountains (*P* = 0.0139). The percentage of *Ae. aegypti* larvae over the total of larvae and pupae for all other aquatic habitats were not significantly associated with daily rainfall (Fig. 1).

## Discussion

The presence of potential aquatic habitats for the proliferation of vector mosquitoes in urban areas is unavoidable, and many of these aquatic habitats such as plastic containers, garbage cans, tires, bromeliads, and buckets are ubiquitous in urban areas around the world^40–42^. Our results are indicating that although *Ae. aegypti* can be found in relatively large numbers in diverse aquatic habitats present in urban areas, nine aquatic habitats were responsible for harboring 80% of all immature *Ae. aegypti* in Miami-Dade County, Florida. Among those aquatic habitats, bromeliads, storm drains, and garbage cans had a significantly lower percentage of *Ae. aegypti* larvae over the total of larvae and pupae adjusted for daily rainfall, indicating that more immature *Ae. aegypti* were able to reach adulthood in these aquatic habitats, in relation to tires, traditionally know as productive aquatic habitats for *Ae. aegypti*.

Other aquatic habitats such as buckets and flower pots are of importance for controlling *Ae. aegypti* population in urban areas, further research should be directed to investigate if these habitats have increased larval competition, thus playing an important role in mosquito development to adulthood. Moreover, buckets and flower pots can be drained and covered more easily than bromeliads and storm drains, making these habitats more transient negatively affecting mosquito development.

On the other hand, our results are indicating that aquatic habitats with lower percentages of *Ae. aegypti* larvae over the total of larvae and pupae adjusted for daily rainfall in relation to tires are exactly the ones that are more difficult to be managed and serviced (i.e., removed or treated). Ornamental bromeliads are widely used in landscaping. These plants naturally accumulate rainwater and draining them to avoid mosquito breeding is not feasible. Insecticide application to treat ornamental bromeliad patches is also challenging since successfully propelling insecticides into all leaf axils and water tanks is an arduous task that has to be done frequently in order to avoid the proliferation of mosquitoes. Storm drains are an integral part of urban areas and are inherently difficult to be managed and serviced to avoid the proliferation of vector mosquitoes^43^. Storm drains not only commonly have large volumes of stagnated water but also have many cryptic habitats in the complex underground tunnel system. However, it has been shown that modifications such as making the base of storm drains higher to allow the stagnated water to drain instead of accumulating can reduce mosquito proliferation in this aquatic habitat^44^.

The number of pupae in a given aquatic habitat has been widely used as a proxy for mosquito production (i.e., the abundance of pupae in an aquatic habitat at a given time) with the objective of determining which of all the potential aquatic habitats are epidemiologically relevant and should be targeted for controlling vector mosquitoes. Such a strategy allows for the identification of the most productive aquatic habitats regardless of its frequency^45,46^.

Even though the result from the tobit model indicated a significant negative association between daily rainfall and the percentage of *Ae. aegypti* larvae over the total of larvae and pupae, the robust regression indicated that only plastic containers and fountains had a significant association with rainfall. This result is an indication that factors other than rainfall or climate are driving the *Ae. aegypti* population dynamics. Human behavior and socioeconomic condition are known drivers for the proliferation of vector mosquitoes^47^. It is not surprising that the aquatic habitats with a lower percentage of *Ae. aegypti* larvae over the total of larvae and pupae adjusted for daily rainfall in relation to tires are intrinsically connected to human actions and behaviors. Ornamental bromeliads used in landscaping require maintenance and are frequently watered, whereas storm drains often have areas constantly flooded with water that are suitable for *Ae. aegypti* proliferation^43^.

In this context, the surveillance of immature mosquito populations in urban areas is essential for guiding mosquito control actions under the IVM framework. The distribution and abundance of vector mosquitoes are driven by complex multi-causal variables, being strongly associated with seasonality. Different species peak in abundance and expand their range in different months of the year depending on their geographic location^48–50^. Moreover, not only can *Ae. aegypti* and other mosquito vector species adapt locally to different features in the urban built environment^51–54^, but they may also exploit the available aquatic habitats differently from region to region. For example, ornamental bromeliads are extensively used in landscaping in Miami and are known for breeding *Ae. aegypti* in great numbers^31,55^, and as highlighted by this study, can have a major role in their proliferation in urban areas. However, in Brazil bromeliads are not considered important for mosquito management and control actions in the cities of Vitória^56^ and Rio de Janeiro^57^, yet are considered important in the city of São Paulo^58^.

Storm drains are increasingly becoming more relevant for the control of *Ae. aegypti* in urban areas^31,43,59^. Our results indicate that not only are a large number of *Ae. aegypti* being produced in storm drains but the number of *Ae. aegypti* reaching the pupal stage is statistically higher when compared to tires. Controlling vector mosquitoes in storm drains is particularly challenging due to the inherent difficulty in reaching all the possible aquatic habitats within the complex underground network of tunnels.

Determining the most important aquatic habitats for *Ae. aegypti* proliferation in urban areas allows the development and implementation of risk thresholds that will help to guide mosquito management and control strategies. Targeting and concentrating control efforts in the most productive containers will render control strategies more effective in decreasing adult *Ae. aegypti* mosquito populations^46,60^. Moreover, the inclusion of other variables such as climate and seasonality can be used for the development of mathematical models to predict fluctuations in the population dynamics of *Ae. aegypti* as well as locations at higher risk of arbovirus transmission. These models can help mosquito control agencies to develop preventative actions and identify and manage potential arbovirus hotspot areas^61,62^.

The presence of highly productive aquatic habitats for *Ae. aegypti* within the household such as bromeliads and garbage cans may lead to an increase in the contact between mosquito vectors and humans leading to a higher risk of arbovirus transmission. Human behavior and socioeconomic conditions should also be considered. Workers that spent a disproportionate amount of time outdoors, such as those in the construction and agriculture workforce, are often more exposed to vector mosquitoes and may be more exposed to arboviruses^63–66^. Therefore, it is essential to remove the most productive aquatic habitats from areas with high concentrations of outdoor workers, such as construction sites, outdoor sports events, and outdoor entertainment areas.

Environmental ordinance is key to control populations of vector mosquito species, and source reduction is an essential element for its effectiveness^20,44^. Determining the most important aquatic habitats in a given area can shed light on the trends of arbovirus transmission risk in urban areas as well as providing important guidelines and new regulations for urban renovations and the urbanization of new areas. Finally, our results can help mosquito control agencies identify where vector mosquitoes are breeding and being produced in higher quantities, and what aquatic habitats should be prioritized in control strategies. Such a framework can lead to the development of more effective preventative strategies that are not only more successful but are also more cost-effective.

As the incidence of arboviral diseases rises globally^67–69^, including in previously non-endemic regions such as in Europe and North America^70–73^, there is a growing need for the development of contingency plans and emergency response guidelines to deal with arbovirus outbreaks. The identification of the most important aquatic habitats for the proliferation of vector mosquito populations in urban areas that should be targeted and prioritized in mosquito control efforts during emergency situations is essential for the development of effective responses to this increasing threat.

The results of this study can greatly help to guide and improve mosquito control operations in Miami-Dade County, Florida. The identification of aquatic habitats in which more *Ae. aegypti* immature specimens can reach adulthood can lead to the development of targeted control strategies prioritizing the controlling efforts in the most productive aquatic habitats. Great results can be achieved towards the reduction of *Ae. aegypti* through community education and engagement on the importance of ornamental bromeliads, storm drains, and garbage cans in the proliferation of *Ae. aegypti*. The results of this study can also positively impact policy, including more effective regulations and guidelines.

Moreover, we were also able to highlight the importance of key aquatic habitats that can be overlooked in day to day mosquito control operations. For example, we were able to show the importance of ornamental bromeliads not only in serving as suitable habitats for *Ae. aegypti* throughout urban areas^31,55^ but also playing a major role in its proliferation in Miami-Dade. These results are of importance since not long ago ornamental bromeliads were not considered suitable aquatic habitats for *Ae. aegypti* in Miami-Dade and were not be considered in control strategies^55^. Only after the Zika virus outbreak in 2016 the importance of ornamental bromeliads in Miami-Dade in supporting the proliferation of *Ae. aegypti* was determined^55^, highlighting the importance of including and prioritizing these plants in mosquito control strategies.

The present study had limitations. The collections were not done in the same aquatic habitat over time as in Wilke 2019^31^, and the immature mosquitoes were not exhaustively collected from all aquatic habitats. It also has to be considered the presence of other cryptic habitats that were undersampled. In this study, we were able to collect immature mosquitoes throughout the incorporated areas of Miami, ranging from rural to highly urbanized areas, thus providing a comprehensive knowledge of how immature *Ae. aegypti* were exploiting different aquatic habitats. However, we were unable to assess the exact contribution of each aquatic habitat to the proliferation of *Ae. aegypti* in areas with different levels of urbanization.

Ultimately, the methodology from this study can be used by mosquito control agencies to identify habitats that should be prioritized in mosquito management and control actions as well as to guide and improve policies and increase community awareness and engagement. The results of this study will allow not only the development of targeted and more effective strategies to control *Ae. aegypti* and other mosquito vector species in urban areas but will also facilitate the development of critical emergency outbreak responses by directing and prioritizing the control response efforts to the most productive aquatic habitats responsible for the maintenance and proliferation of vector mosquitoes in urban areas.

## Methods

### Study area

Here we focus on Miami-Dade County, Florida. Miami-Dade is the most populous county in Florida and serves as a major gateway to the United States to people coming and going from South and Central America as well as from the Caribbean region^74,75^. Miami was the most affected county in the continental United States during the Zika virus outbreak in 2016^76^. Moreover, 212 imported and 14 locally acquired dengue cases were reported in 2019 in Miami, causing the Florida Department of Health to issue a mosquito-borne illness alert^77,78^.

Miami has the ideal conditions for the proliferation of vector mosquitoes. Since the Zika virus outbreak in 2016 the Miami-Dade Mosquito Control Division capacity has been greatly enhanced. As part of the effort to better understand how to better control populations of vector mosquito species in Miami, an adult mosquito surveillance system has been in place since August 2016. This system provides valuable insight into the population dynamics of vector mosquitoes in Miami^48^. Moreover, many studies have been conducted in areas considered problematic for controlling vector mosquitoes in Miami such as construction sites^79^, tire shops^80^, ornamental bromeliad patches^55^, cemeteries^81^, and urban farms^66^. Together, these studies have provided key evidence of vector mosquito distribution in Miami.

In an effort to better understand how vector mosquitoes, mainly *Ae. aegypti,* are distributed among different aquatic habitats we have, alongside with the Miami-Dade Mosquito Control Division, established an immature mosquito surveillance system based on requested by citizen complaints through 311 calls^31^. The majority of the complaint calls are motivated by the presence of adult mosquitoes acting as a great indicator for the presence of immature breeding in the area. Building on the results obtained by this surveillance system, which elucidated the most common aquatic habitats for *Ae. aegypti* as well as their distribution in Miami^31^, we aimed to determine what are the most important aquatic habitats to be prioritized and targeted by future mosquito control strategies.

### Mosquito inspections

**Figure 2:**
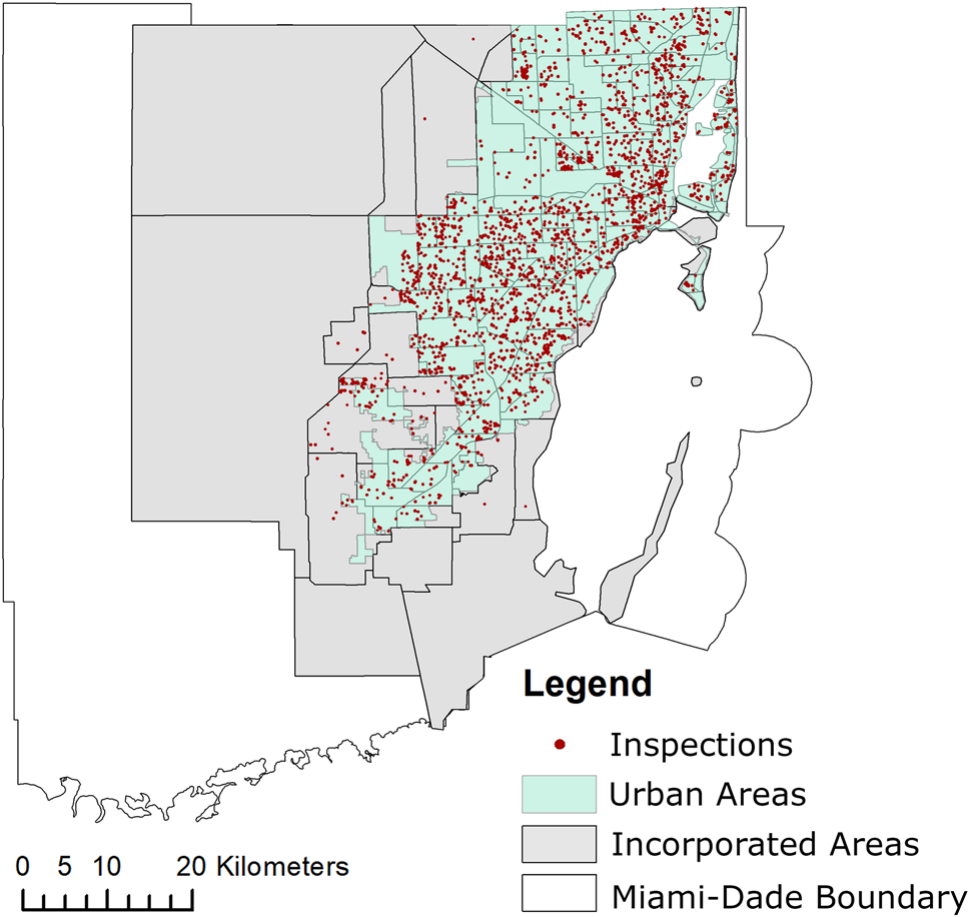
Map showing the location of the mosquito inspections in Miami-Dade, Florida from June 1st, 2018 to October 31st, 2019. The red dots represent the point source location of the 311 complaint calls that were investigated for potential sources for mosquito breeding and development in a 50-m radius from the complaint location. Figure was produced using ArcGIS 10.2 (Esri, Redlands, CA) using freely available layers from the Miami-Dade County’s Open Data Hub—https://gis-mdc.opendata.arcgis.com/.

A total of 3,354 inspections were conducted in Miami-Dade County, Florida from June 1st, 2018 to October 31st, 2019 as in Wilke et al.^31^. Surveys were made in response to citizen requests through 311 calls which automatically created a Service Request (SR). Based upon the SR, a Mosquito Control Inspector was dispatched within 48 h to assess and investigate any potential sources for mosquito breeding and development in a 50-m radius from the complaint location. Since inspections were triggered by citizens' requests, they were initially made on private properties both indoors and outdoors at the discretion of the owner or responsible adult. Public spaces within the 50-m radius from the complaint location were also surveyed and included in this study. The 311 calls are considered exceptionally informative since they represent precise locations in which residents needed assistance in controlling mosquitoes (Fig. 2).

### Mosquito collection

At each inspection, the Miami-Dade Mosquito Control inspectors collected the immature mosquitoes using entomological dippers and manual plastic pumps (turkey basters). The immature mosquitoes collected in each aquatic habitat within a radius of 50 m from the original point source location of the 311 calls were then held in plastic bags (100 ml), as in Wilke et al.^31^ and transported to the Miami-Dade County Mosquito Control Laboratory. Larvae were allowed to develop for 24 h to increase the reliability of the identification and pupae were allowed to emerge as adults and then identified. All specimens were morphologically identified to species using taxonomic keys^82^.

Since this study posed less than minimal risk to participants and did not involve endangered or protected species the Institutional Review Board at the University of Miami determined that the study was exempt from institutional review board assessment (IRB Protocol Number: 20161212).

### Statistical analysis

We used *Ae. aegypti* larvae and pupae count data, type, and count of aquatic habitats, daily maximum and minimum temperature, and daily rainfall obtained from the National Weather Services (available at: https://www.weather.gov/mfl/). First, we calculated the percent of larvae over the percent of larvae and pupae. Therefore, a lower percent of larvae over the percent of larvae and pupae indicates the presence of a higher rate of pupae in relation to larvae. We opted to use the percent of larvae over the percent of larvae and pupae as the dependent variable since it can provide meaningful indications of how the population dynamics of *Ae. aegypti* is associated with the different conditions of each aquatic habitat as well as to climate conditions. This approach is useful to assess habitat quality, in which a low percentage of larvae (i.e., a high percentage of larvae and pupae) show that a substantial number of immature mosquitoes in a given aquatic habitat will be reaching the adult stage.

Aquatic habitats such as ponds and canals, in which *Ae. aegypti* was completely absent were not included in the analysis. However, on many occasions, larvae were found breeding in a given aquatic habitat but no pupae were found, as well as the other way around. In that case, the absence of either larvae or pupae was considered as 0 instead of no-data.

Then we used backward stepwise tobit regression with a lower bound of the percentage of larvae to total larvae and pupae and ran a backward selection model and removed variables that were not statistically significant until just rainfall and habitat type were significant^83^. The Ordinary Least Squares regression (OLS) coefficients from the tobit model are useful to assess the difference in larvae pupae ration by habitats^84^. The tobit regression model was used since mosquito count data is intrinsic zero-inflated and censoring the dependent variable is advisable to mitigate this issue. We opted to use the tobit regression model since it would allow us to limit the measures as a percentage from 0 to 100%. Then, those thresholds were used in the model specification as the cutoffs (0,1) so that the model would not make estimates outside of these regions.

Tires are especially conducive to the proliferation of *Ae. aegypti*, immature mosquitoes can hide from predators and the rubber from which tires are made of provides efficient thermal insulation from the elements providing optimum resting places. Therefore, due to the known association between the availability of tires and the proliferation of *Ae. aegypti* in urban areas, tires were used as the standard to assess the output of all the other aquatic habitats^80,85–87^.

To assess the association between daily rainfall and the larvae pupae ratio by habitat we used a robust regression. The robust regression uses an iteratively re-weighted least squares method and Huber weights. As a result, a small number of influential observations would not strongly affect the estimate of the linear association between daily rainfall and the larvae pupae ration within each habitat.
